# Artesunate potentiates antibiotics by inactivating heme-harbouring bacterial nitric oxide synthase and catalase

**DOI:** 10.1186/1756-0500-4-223

**Published:** 2011-06-30

**Authors:** Qing-Ping Zeng, Na Xiao, Pei Wu, Xue-Qin Yang, Li-Xiang Zeng, Xiao-Xia Guo, Ping-Zu Zhang, Frank Qiu

**Affiliations:** 1 Tropical Medicine Institute, Guangzhou University of Chinese Medicine, Guangzhou, China; 2 Artemisinin Research Centre, Guangzhou University of Chinese Medicine, Guangzhou, China; 3 Simplex Biotechnologies, LLC, Clinton, NJ08809, USA

## Abstract

**Background:**

A current challenge of coping with bacterial infection is that bacterial pathogens are becoming less susceptible to or more tolerant of commonly used antibiotics. It is urgent to work out a practical solution to combat the multidrug resistant bacterial pathogens.

**Findings:**

Oxidative stress-acclimatized bacteria thrive in rifampicin by generating antibiotic-detoxifying nitric oxide (NO), which can be repressed by artesunate or an inhibitor of nitric oxide synthase (NOS). Suppressed bacterial proliferation correlates with mitigated NO production upon the combined treatment of bacteria by artesunate with antibiotics. Detection of the heme-artesunate conjugate and accordingly declined activities of heme-harbouring bacterial NOS and catalase indicates that artesunate renders bacteria susceptible to antibiotics by alkylating the prosthetic heme group of hemo-enzymes.

**Conclusions:**

By compromising NO-mediated protection from antibiotics and triggering harmful hydrogen peroxide burst, artesunate may serve as a promising antibiotic synergist for killing the multidrug resistant pathogenic bacteria.

## Findings

**Figure 1:**
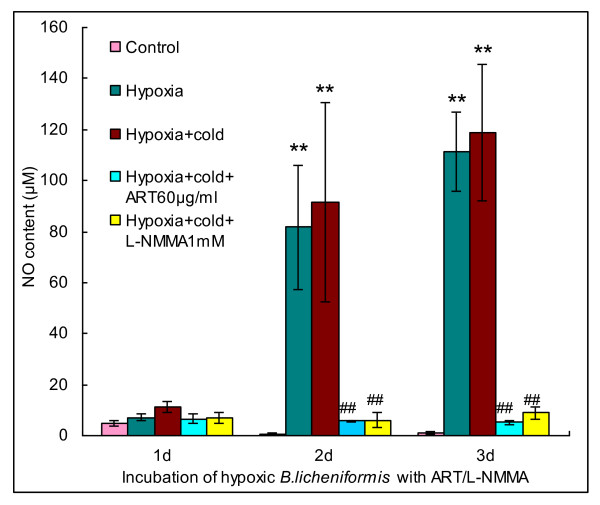
**Production of NO from *B. licheniformis *upon acclimatization to oxidative stress and suppression of oxidative stress-inducible NO as incubation of *B. licheniformis *with artesunate or a NOS inhibitor**. ART: artesunate; L-NMMA: NG-monomethyl-L-arginine monoacetate. Double asterisks (**) represent very significant difference from the control (*P *< 0.01); Double wells (##) represent very significant difference from the hypoxia + cold group (*P *< 0.01).

Artesunate is a semi-synthetic soluble derivative of artemisinin, a plant sesquiterpene endoperoxide lactone with pleiotropic functions of anti-malaria, anti-tumour and anti-inflammation [1]. Here, we report that artesunate can also exert an accelerated anti-bacterial activity in combination with antibiotics. Initially, we found that artesunate mitigated the oxidative stress-induced generation of nitric oxide (NO) from the Gram-positive bacterium, *Bacillus licheniformis*. A gradual increase of nitrate/nitrite, the oxidation product of NO, was detected in the bacterial culture standing without agitation either at room temperature (the hypoxia group) or in a 4°C refrigerator (the hypoxia + cold group) although no significant difference was observed between the hypoxia + cold group and the hypoxia group (Figure 1). In similar, it has been demonstrated that NO is not consumed and accumulates in the microenvironment of human tissue at lower oxygen concentrations [2]. It is also manifested from Figure 1 that oxidative stress-triggered NO generation was repressed by either artesunate or a specific inhibitor of nitric oxide synthase (NOS), NG-monomethyl-L-arginine monoacetate (L-NMMA), indicating that artesunate may represent a novel NOS inhibitor that acts in an unknown manner.

**Figure 2:**
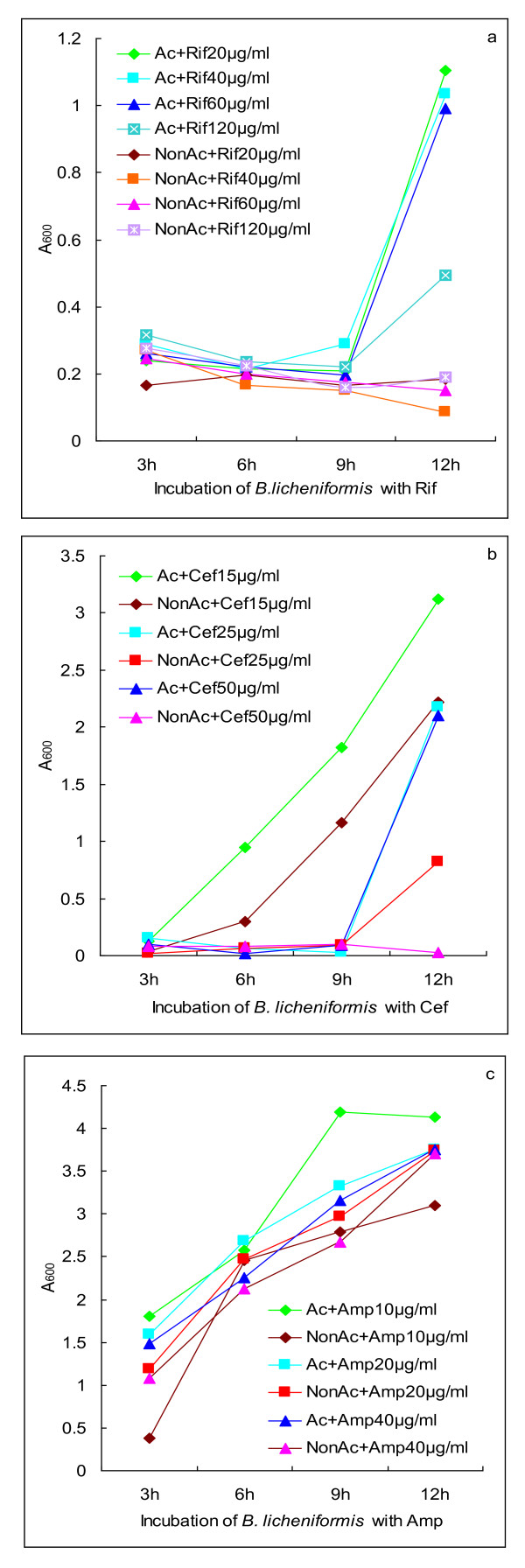
**Inducible NO-mediated protection of *B. licheniformis *from antibiotics**. (a) Propagation of acclimatized or non-acclimatized *B. licheniformis *in the presence of rifampicin. (b) Propagation of acclimatized or non-acclimatized *B. licheniformis *in the presence of cefotaxime. (c) Propagation of acclimatized or non-acclimatized *B. licheniformis *in the presence of ampicillin. Ac: acclimatization to hypoxia; NonAc: non-acclimatization to hypoxia. Rif: rifampicin; Cef: cefotaxime; Amp: ampicillin.

Knowing that bacterial NO can detoxify antibiotics through direct or indirect mechanisms [3], we tested whether NO that was triggered by acclimatization to hypoxia confers *B. licheniformis *tolerance to the antibiotic rifampicin. Consequently, when bacteria propagated to a logarithmic phase in the absence of rifampicin, acclimatized bacteria started to proliferate at a higher rate than non-acclimatized bacteria in the presence of rifampicin (Figure 2a). Similarly, acclimatized bacteria also showed somewhat accelerated proliferation in the presence of either cefotaxime (Figure 2b) or ampicillin (Figure 2c).

**Figure 3:**
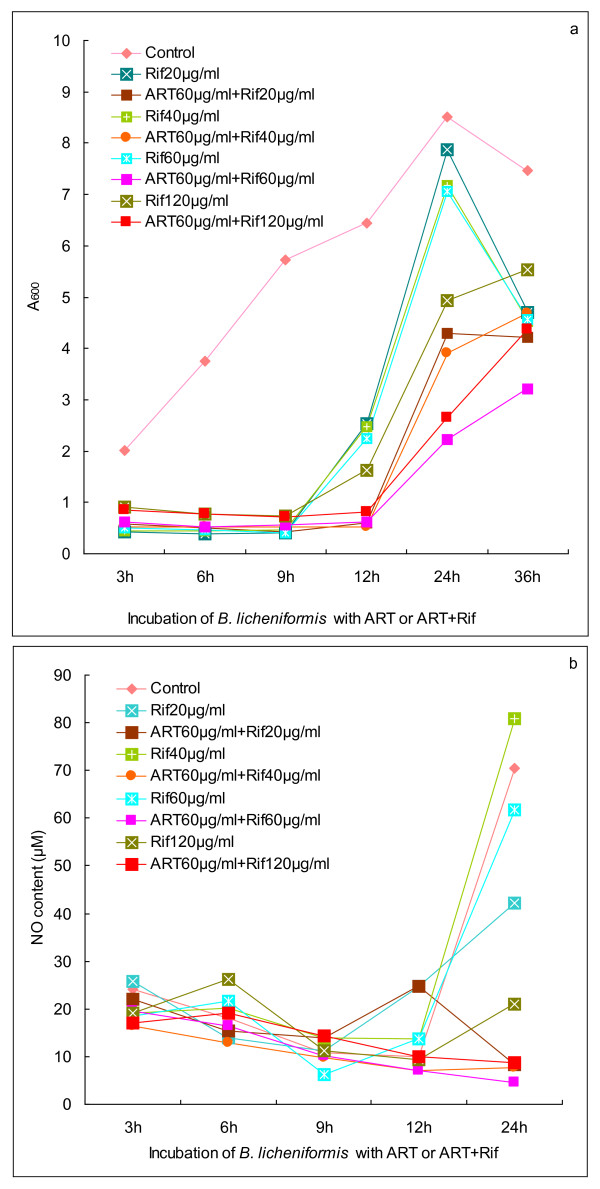
**Bacterial propagation and NO generation in the presence of antibiotics or artesunate + antibiotics for *B. licheniformis***. (a) Propagation of *B. licheniformis *in the presence of rifampicin or artesunate + rifampicin. (b) Generation of NO from *B. licheniformis *in the presence of rifampicin or artesunate + rifampicin. Rif: rifampicin; ART: artesunate.

Due to attenuating protective NO production in bacteria, artesunate was anticipated to reverse NO-mediated protection of bacteria from antibiotics. Indeed, artesunate in combination with rifampicin led to more stringent growth inhibition of *B. licheniformis *than rifampicin alone. As illustrated in Figure 3a, much slower growth of bacteria was monitored in artesunate + rifampicin than in rifampicin, hence addressing that artesunate potentiates rifampicin perhaps by attenuating NO production from bacteria. In the present study, we observed that enhanced NO burst occurred in bacteria that were exposed to rifampicin, whereas less NO was measured in bacteria that were co-treated by artesunate with rifampicin (Figure 3b).

**Figure 4:**
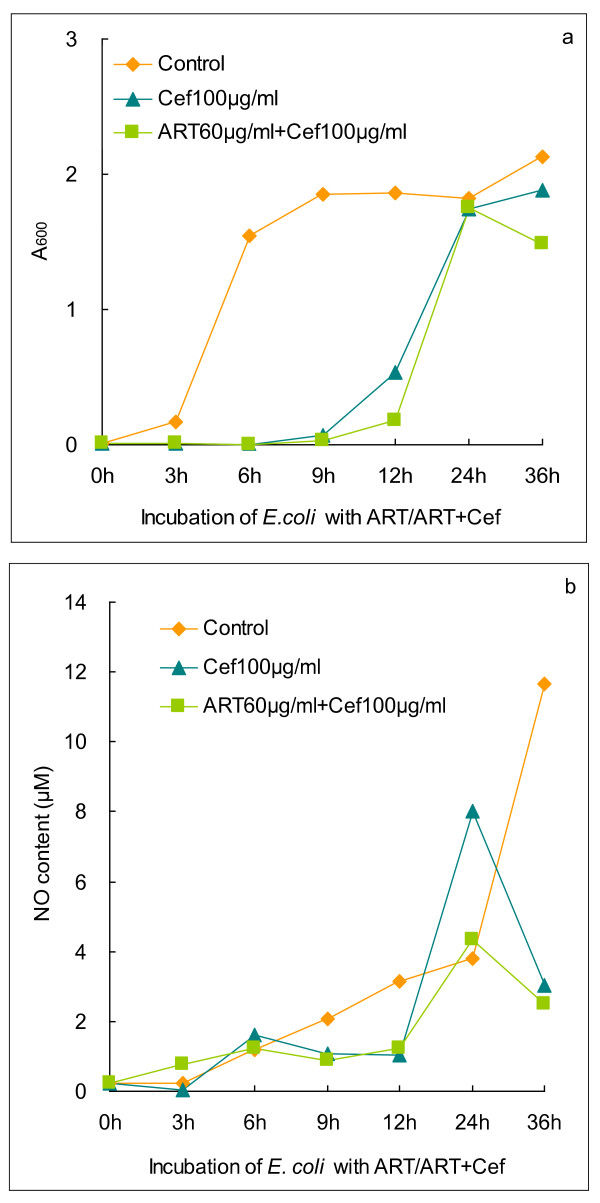
**Bacterial propagation and NO generation in the presence of antibiotics or artesunate + antibiotics for *E. coli***. (a) Propagation of *E. coli *in the presence of cefotaxime or artesunate + cefotaxime. (b) Generation of NO from *E.coli *in the presence of cefotaxime or artesunate + cefotaxime. Cef: cefotaxime; ART: artesunate.

In the NOS-free Gram-negative bacterium, *Escherichia coli*, a complex compassing nitrite reductase, flavohemoglobin and a NO-sensing regulator is harnessed to form NO [4]. Indeed, we detected the generation of NO in *E. coli*, either in the presence or absence of cefotaxime, but no correlation of bacterial proliferation with NO production was established (Figure 4a and 4b). From the extremely low level of NO in *E. coli*, we inferred that such a trace amount of NO might insufficient to sensitize cefotaxime.

**Figure 5:**
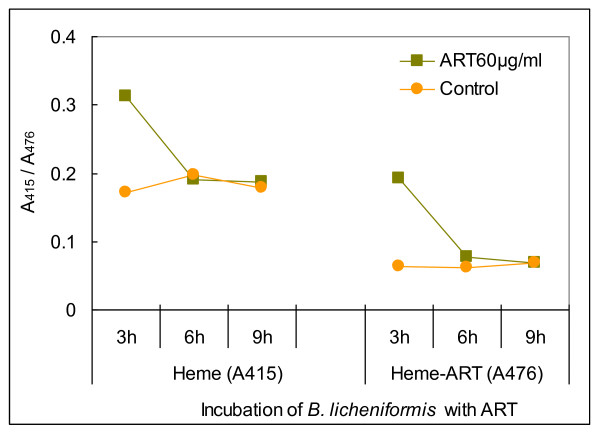
**Time-course monitoring of the heme-artesunate conjugate in *B. licheniformis *incubated with artesunate**. ART: artesunate. A_415 _represents the absorbance of heme at the wavelength of 415 nm; A_476 _represents the absorbance of the heme-artesunate conjugate at the wavelength of 476 nm.

The non-pathogenic *Bacillus subtilis *and pathogenic *Bacillus anthracis *as well as many other Gram-positive bacteria generate NO by their own NOS that associates with a prosthetic heme group for the reduction of ferric heme [Fe^3+^] to ferrous heme [Fe^2+^] [5]. Given that heme alkylation by artemisinin has been verified by identifying the heme-artemisinin adduct in malaria-infected mice [6], and the interaction between heme and artesunate has been also recognised by monitoring the dynamic shift of one peak specific to heme and another peak unique to the heme-artesunate conjugate in tumour cells [7], we assumed that artesunate would also bind to the heme group of bacterial NOS and hence prohibit the inter-conversion of Fe^3+ ^with Fe^2+ ^within the hemoprotein. Indeed, A_415 _that reflects the absorbance of heme and A_476 _that represents the absorbance of the heme-artesunate conjugate reached high values after the bacterial culture was supplemented with artesunate for 3 h (Figure 5), suggesting that the A_415 _peak was likely resulted from the increase of NOS, while the A_476 _peak was probably derived from the conjugation of heme with artesunate. Nevertheless, the increase of NOS needs the overexpression of a corresponding gene that encodes NOS in *B. licheniformis*, whether *NOS *gene is inducible by artesunate-bound inactivation of NOS awaits further elucidation.

**Figure 6:**
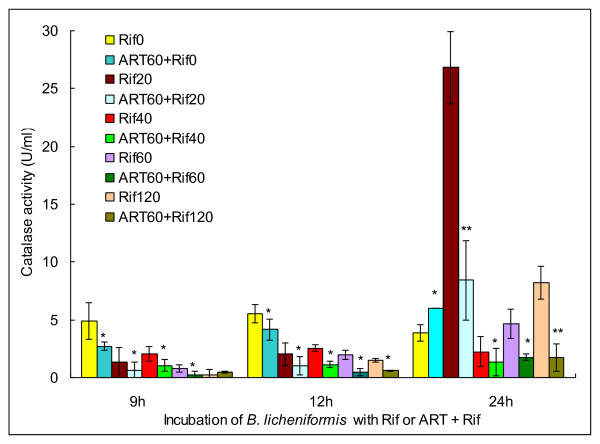
**Determination of catalase activity in *B. licheniformis *upon exposure to rifampicin or artesunate + rifampicin. ART: artesunate; Rif: rifampicin**. A single asterisk (*) represents significant difference of the artesunate + rifampicin group from the rifampicin group (*P *< 0.05); Double asterisks (**) represent very significant difference of the artesunate + rifampicin group from the rifampicin group (*P *< 0.01).

Until recently, there has no documented evidence regarding the impact of artesunate on bacterial catalase. Considering that catalase is also a heme-harbouring enzyme among *Bacillus *[8], it can be deduced that a similar mechanism exists in *B. licheniformis*, by which artesunate binds to the prosthetic heme group of catalase and abrogates the conversion of hydrogen peroxide. Therefore, we further measured catalase activity after incubating bacteria with rifampicin or rifampicin + artesunate. The results showed that enzyme activity was considerably reduced once artesunate was included in the culture (Figure 6). In other observations, NO activates catalase in bacteria by diminishing the rate of cystine reduction to cysteine, which drives the Fenton reaction and simultaneously inhibits catalase [9]. Due to catalase inhibition, hydroxyl radicals that were derived from excess hydrogen peroxide exhibit the extreme toxicity to bacterial DNA through base modifications and strand breaks [10]. It can be concluded that artesunate facilitates the action of antibiotics against bacteria by synchronously inactivating NOS and catalase. Interestingly, artesunate also covalently binds to hemoproteins of tumour cells and potentiates the cytotoxicity of 5-fluorouracil *in vitro *and *in vivo *[11], implying that a common mechanism exists to interfere with the fate of bacterial and tumour cells. However, inhibition of a bacterial multidrug efflux pump system should represent an alternative manner of artesunate in sensitizing antibiotics [12].

A current challenge of coping with bacterial infection is that bacterial pathogens are becoming less susceptible to or more tolerant of commonly used antibiotics. For example, globally endemic tuberculosis caused by multidrug resistant strains of *Mycobacterium tuberculosis *remains a formidable threat to human health. Although the development of more potent antibiotics may prohibit the lethal pathogens from worldwide transmission, it has proved to be costly, time-consuming and technically difficult [13]. Otherwise, if an antibiotic synergist like artesunate can benefit to fight against the antibiotic resistant bacteria, it will minimise the dosage of antibiotics in antibacterial therapy and diminish the heavy incidence and rapid transmission of multidrug resistant bacterial pathogens.

## Methods

### Bacterial culture and NO content estimation

A single colony of *B. licheniformis *BL20386 or *E. coli *DH5α was inoculated in LB broth and cultured overnight at 37°C. A 1% aliquot of pre-cultured bacteria was inoculated in LB broth and cultured at 37°C until absorbance at 600 nm (A_600_) to 0.6. For oxidative stress acclimatization, triangle bottles with the bacterial culture were placed without agitation for 3 d at room temperature (the hypoxia group) or in a 4°C refrigerator (the hypoxia + cold group). For antibiotic exposure, a 1% aliquot of the overnight bacterial culture was inoculated in LB broth supplementing with different concentrations of rifampicin, cefotaxime, or ampicillin. For inhibitor treatment, a 1% aliquot of the overnight bacterial culture was inoculated in LB broth supplementing with 60 μg/ml artesunate or 1 mM NG-monomethyl-L-arginine monoacetate (L-NMMA). On each day, 1 ml of the bacterial culture was taken out for estimation of NO content using a commercially available kit and according to the manufacturer's instruction. The content of NO is represented by the amount of nitrate (NO_3_^-^)/nitrite (NO_2_^-^). For plotting a standard curve of nitrate/nitrite, 1 M NaNO_3 _was dissolved to a series of dilutions (1, 2, 5, 10, 20, 30, 40, 60 μM) by LB broth for measurement of A_540_, from which a regression equation and a determinant coefficient were calculated.

### Bacterial growth assay

For monitoring the growth rate of oxidative stress-acclimatized bacteria, a 1% aliquot of the bacterial culture standing at room temperature for 3 d or that without oxidative stress treatment was inoculated in LB broth supplementing with rifampicin and cultured at 37°C with agitation. After cultured for 3, 6, 9 and 12 h, 1 ml of the bacterial culture was taken out and diluted with 9 ml of fresh LB broth to measure A_600 _for plotting the growth rate curve. For assaying the bacterial growth rate following treatment by rifampicin or artesunate + rifampicin, a 1% aliquot of the overnight bacterial culture was inoculated in LB broth supplementing with 60 μg/ml artesunate and different concentration of rifampicin, and cultured at 37°C. After cultured for 3, 6, 9, 12 and 24 h, 1 ml of the bacterial culture was diluted with 9 ml of fresh LB broth to measure A_600 _for plotting the growth rate curve.

### Detection of heme and heme-artesunate conjugate

A 1% overnight culture of *B. licheniformis *BL20386 was inoculated in LB broth supplementing with 60 μg/ml artesunate and cultured at 37°C for 2 d. The triangle bottle with a pre-culture was first placed at ambient temperature for 1 d and subsequently at a 4°C refrigerator for 1 d. After supplementing with fresh LB broth and 60 μg/ml artesunate, the bacterial strain was continuously cultured overnight at 37°C. The culture was taken out on 3, 6 and 9 h for collecting cells by centrifugation and lysing them through repeated cycles of frozen-thaw, and finally the lysate was applied to measure A_415 _and A_476_.

### Determination of catalase activity

A 1% overnight culture of *B. licheniformis *BL20386 was inoculated in LB broth supplementing with 60 μg/ml artesunate in the treatment group or without artesunate in the control group, and cultured at 37°C for 2 d. The triangle bottle with a pre-culture was first placed at ambient temperature for 1 d and subsequently at a 4°C refrigerator for 1 d. After supplementing with fresh LB broth containing 60 μg/ml artesunate or 60 μg/ml artesunate + different concentrations of rifampicin, the bacterial strain was continuously cultured overnight at 37°C. The culture was taken out from each group on 9, 12 and 24 h for measurement of A_405 _using a commercially available kit. The activity of catalase (U/ml) was calculated according the formula of (A_405 control group _- A_405 treatment group_) × 271 × 1/6.

## Competing interests

The authors declare that they have no competing interests.

## Authors' contributions

NX, PW and XQY were responsible for bacterial culture and drug evaluation. LXZ and XXG carried out the spectrophotometric assay. PZZ performed the statistical analysis. QPZ designed and coordinated the experiment. FQ helped to draft the manuscript. All authors read and approved the final manuscript.
